# High failure rate but promising clinical performance after implantation of a flexible medial meniscus prosthesis at 1‐year follow‐up

**DOI:** 10.1002/ksa.12454

**Published:** 2024-09-03

**Authors:** Branco S. van Minnen, Petra J. C. Heesterbeek, Koen C. Defoort, Pieter J. Emans, Ewoud R. A. van Arkel, Thijmen Struik, Liesbeth M. Jutten, Saskia Susan, Sebastiaan A. W. van de Groes, Nico Verdonschot, Tony G. van Tienen

**Affiliations:** ^1^ Radboud University Medical Centre Nijmegen The Netherlands; ^2^ ATRO Medical B.V. Uden The Netherlands; ^3^ Sint Maartenskliniek Nijmegen Ubbergen The Netherlands; ^4^ Maastricht University Medical Centre Maastricht The Netherlands; ^5^ Haaglanden Medical Centre Den Haag The Netherlands

**Keywords:** artificial meniscus, clinical investigation, meniscus prosthesis, meniscus replacement, postmeniscectomy pain syndrome

## Abstract

**Purpose:**

After unsatisfactory results in a first‐in‐human clinical investigation with an anatomically shaped medial meniscus prosthesis, the prosthesis and its fixation technique were altered. This interim analysis of a prospective single‐arm clinical investigation aims to evaluate safety and clinical performance in a first‐in‐human study with the redesigned meniscus prosthesis system.

**Methods:**

Ten patients suffering from medial postmeniscectomy pain syndrome were treated with the meniscus prosthesis. Patient‐reported outcome measures were obtained at baseline and at 6‐week, 3‐, 6‐, and 12‐month follow‐up. Radiographs and magnetic resonance imaging scans were obtained to evaluate joint degeneration and prosthesis location.

**Results:**

The device alterations resolved the issues occurring with the previous design, but four prostheses were explanted after fixation failure or subluxation of the prosthesis. Five out of six patients who reached 1‐year follow‐up reported a clinically significant improvement of the knee injury and osteoarthritis outcome score pain subscale. Imaging revealed no adverse effects on joint degeneration.

**Conclusion:**

The failure mechanisms that occurred with the previous design have been resolved, but the new fixation technique introduced new safety issues. Improvement of positioning and fixation techniques are considered essential future adaptations to reduce the risk of failure. The good clinical outcomes reported by the patients reaching 1‐year follow‐up indicate that the medial meniscus prosthesis is a potential solution for patients suffering from postmeniscectomy pain syndrome.

**Level of Evidence:**

Level II.

**Abbreviations**
CI: confidence interval
ESUG: Early Safety User Group
KOOS: Knee injury and osteoarthritis outcome score
MAT: meniscal allograft transplantation
MCID: minimal clinically important difference
OA: osteoarthritis
OKS: Oxford knee score
PCU: polycarbonate polyurethane
PROM: patient‐reported outcome measure
WORQ: Work, osteoarthritis, and joint‐replacement questionnaire

## INTRODUCTION

The postmeniscectomy pain syndrome can be described as a dull and nagging pain, often occurring after a pain‐free interval after partial or total meniscectomy. Damage or absence of (part of) the meniscus may be accompanied by deterioration of the articular cartilage and onset of degeneration [13]. If conservative treatment is insufficient and the postmeniscectomy pain is accompanied by knee malalignment, a correction osteotomy is typically preferred [2]. Patients without malalignment who underwent partial meniscectomy may benefit from meniscal scaffolds, for example, Actifit® or the Collagen Meniscal Implant, although the quality of available clinical evidence is limited [8]. Meniscal allograft transplantation (MAT) is an established surgical solution after total meniscectomy, demonstrating improved patient‐reported outcome measures (PROMs) even after long‐term follow‐up [9].

However, the technical difficulty of MAT, logistic challenges and the lack of available size‐matched allografts give rise to the need for artificial total meniscus replacements [5, 14]. NUsurface® is a subtotal medial meniscus implant, requiring an intact meniscus rim of at least 2 mm to avoid dislocation [11]. The short‐term clinical outcomes are promising, but the lack of tibial fixation still resulted in a relatively high dislocation rate [26]. Furthermore, the non‐anatomical shape sometimes requires a notchplasty to fit and might contribute to a relatively high rate of implant ruptures [20, 26].

The Artimis® meniscus prosthesis, previously known as Trammpolin®, was designed with an anatomical shape and tibial fixation as an alternative to MAT. The anatomical shape of the medial meniscus was obtained by calculating the average geometry of 35 healthy menisci from MR images [24]. The first design of this artificial meniscus prosthesis consisted of a meniscus body with two horns for fixation at the anatomical meniscus attachment sites and a reinforced core to withstand circumferential forces [21]. The horns and core were made of a relatively stiff polycarbonate polyurethane (PCU), while the cartilage‐contacting surface was made of a softer variant of the same PCU. Two titanium screws were used to rigidly fix the prosthesis horns to the tibial plateau. In a first‐in‐human clinical study, a small cohort of patients with medial postmeniscectomy pain was implanted with the meniscus prosthesis, and the results were published in this journal [21]. After five patients, inclusion was discontinued due to several adverse events. Device‐related events occurred in all patients and included joint effusion, range of motion (RoM) deficit, persistent pain, and implant failure. As a result, four prostheses were removed within 12 months. Positive findings were an unchanged cartilage status and a return to preoperative levels of PROMs after explantation. Moreover, the remaining patient performed excellent up to 2 years after implantation. Nonetheless, the first version of the meniscus prosthesis did not perform as well as anticipated, and redesign of the prosthesis, its fixation method and the surgical technique were deemed necessary.

**Figure 1:**
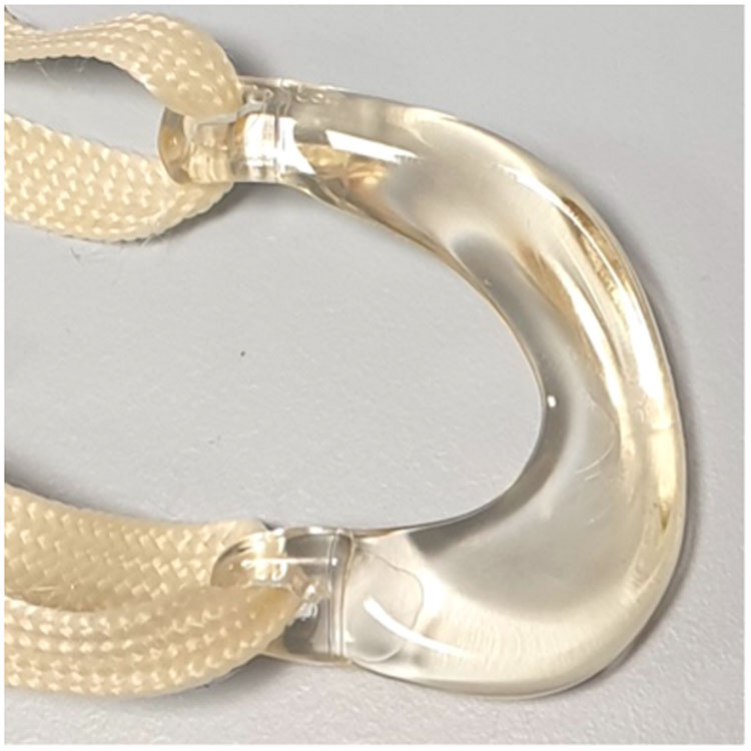
The Artimis® medial meniscus prosthesis.

It was hypothesized that device breakage was mainly caused by the relatively high stiffness of the meniscus prosthesis and its fixation technique [21]. To enable better adaptation to the joint geometry and movements, the stiff core was replaced by a more flexible meniscus body and a less rigid fixation technique was developed (Figure 1) [12]. Preclinically, it was demonstrated that this second‐generation meniscus prosthesis allowed more load‐sharing between the prosthesis and the direct tibiofemoral contact [12]. As less load is transferred through the meniscus prosthesis and its fixation points, reduced internal stresses and lower failure rates were expected. The increased ability to deform and move over the tibial plateau should prevent impingement and subsequent RoM deficit and allow for small deviations from optimal positioning. Moreover, this biomechanical study showed that the flexible prosthesis is still capable of significantly reducing contact pressures in the meniscectomized knee joint.

It was therefore hypothesized that this redesigned meniscus prosthesis and its fixation are able to withstand the physiological knee joint loads and that it may be beneficial for patients with medial postmeniscectomy pain. The purpose of this first‐in‐human clinical study was to assess the safety and clinical performance of the updated meniscus prosthesis system, including its fixation technique and surgical procedure. Furthermore, it aimed to provide insights on the resulting effect on the experienced postmeniscectomy pain and other clinical outcomes to assess clinical efficacy. This interim analysis evaluated all available data of the ongoing clinical study in order to identify any device‐related adverse events at an early stage and to reveal whether a proof‐of‐concept can be clinically demonstrated after 1‐year follow‐up.

## MATERIALS AND METHODS

This interim analysis at the 1‐year follow‐up is part of the AIR2 study, which is a 2‐year prospective single‐arm, multicentre clinical investigation. The study is designed as a feasibility study to demonstrate safety and clinical performance. Based on the previous study, a sample size of 10 patients was deemed appropriate to provide an estimate of the occurrence of device‐related adverse events and an initial evaluation of clinical efficacy.

The main inclusion and exclusion criteria are listed in Table 1; the complete set can be found on www.toetsingonline.nl, using the CCMO ID.

**Table 1:** Summary of inclusion and exclusion criteria.

Inclusion criteria	Exclusion criteria
(1) Medial compartment knee pain (2) Medial partial or total meniscectomy (3) >6 months ago (4) KOOS Pain ≤75 (100 being no pain) (5) Aged ≥18 and ≤70 years (6) BMI ≤ 30 kg/m^2^ (7) Knee alignment: <5° varus or valgus alignment (8) Willing to be implanted with the meniscus prosthesis (9) Willing and able to comply to follow‐up visits, questionnaires and imaging (10) Willing and able to understand and sign the informed consent form (11) Able to read and understand the Dutch language	(1) Symptomatic knee due to a tear that might be addressed by a repeat meniscectomy (2) Modified Outerbridge grade 4 cartilage loss in the medial compartment, potentially contacting the meniscus prosthesis (3) Pain and modified Outerbridge grade 3 or 4 cartilage score in the lateral and/or patellar compartment (4) Laxity level of more than Grade II (IKDC), primary or secondary to a ligament injury (5) Significant trochlear dysplasia, patellar instability, or symptomatic patellar misalignment (6) Obvious evidence of medial femoral squaring (7) ACL reconstruction <9 months ago (8) Active infection, tumour, or osteoporosis

Abbreviations: ACL, anterior cruciate ligament; BMI, body mass index; IKDC, International Knee Documentation Committee; KOOS, knee injury and osteoarthritis outcome score.

The principal investigators constituted the Early Safety User Group (ESUG). ESUG meetings were scheduled regularly to evaluate usability, safety, and recommendations regarding the surgical technique. A data monitoring committee was established to provide independent advice on patient safety and the scientific validity of the study.

### Outcome measures

The main purpose of this interim analysis is to evaluate device‐related adverse events and to assess whether new failure modes occur compared to those identified in the previous first‐in‐human study.

This interim analysis further includes clinical outcomes preoperatively and after follow‐up of 6 weeks, 3, 6 and 12 months after surgery. PROMs collected at these timepoints were all knee injury and osteoarthritis outcome score (KOOS) subscales, visual analogue scale pain score, Lysholm score, Oxford knee score (OKS), 5‐level EQ‐5D health utility score, and work, osteoarthritis, and joint‐replacement questionnaire (WORQ). The change (Δ) in the KOOS Pain subscales after 12 months was defined as the primary clinical endpoint of this interim analysis. For this outcome measure, the minimal clinically important difference (MCID) is defined as an increase of 9.9 points [10].

At baseline and after 12 months, weight‐bearing knee radiographs were obtained to evaluate the severity of osteoarthritis (OA) by Kellgren–Lawrence classification. Modified Outerbridge grading was performed on magnetic resonance imaging (MRI) scans to evaluate cartilage status at these timepoints. At the 12‐month follow‐up, the medial protrusion of the prosthesis was evaluated by measuring the distance between the medial edge of the meniscus prosthesis and the tibial plateau, on the coronal image corresponding to the apex of the medial tibial spine [4]. Furthermore, the postoperative MR images allowed for the assessment of prosthesis integrity and a retrospective evaluation of the location of the fixation points. All analyses were performed by the principal investigators according to standardized methods but without evaluation of intra‐ and interobserver variability.

### Meniscus prosthesis system

**Figure 2:**
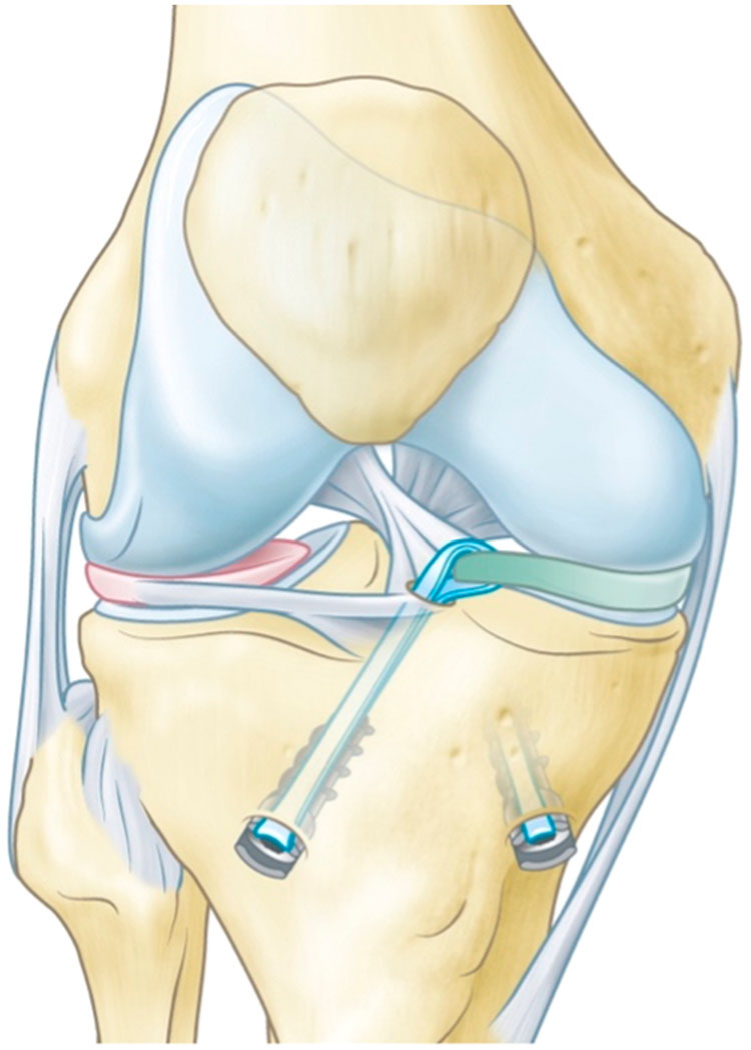
The meniscus prosthesis and its tibial fixation.

The Artimis® medial meniscus prosthesis system is designed by ATRO Medical (Nijmegen, The Netherlands). The meniscus prosthesis is made of Bionate® (DSM), a PCU well known for its biocompatibility and low friction with cartilage [6, 22]. The cartilage‐contacting middle section, or body, has an anatomical shape and is made of the soft and flexible Bionate® II 80A, while the fixation horns are made of the stiffer and stronger Bionate® 75D (Figure 1). Five different sizes for the left and right knee are manufactured by injection moulding and sterilized with ethylene oxide. Fixation to the anatomical meniscus attachment sites on the tibial plateau is achieved by fixation tapes made of polyethylene terephthalate, which are anchored in two bone tunnels with polyether ether ketone interference screws (Figure 2).

### Surgical technique

Implantation of the meniscus prosthesis was performed by three orthopaedic surgeons with experience in knee arthroscopy, including ACL reconstruction and meniscus surgery (K. C. D., P. J. E. and E. R. A. v. A.). Under anaesthesia, the surgeon assessed knee stability and preoperative RoM. Medial and lateral parapatellar arthroscopic portals were made for a standard arthroscopic approach, the indications for implantation were confirmed and the medial collateral ligament was released if needed to increase posterior horn exposure and accessibility. The remaining parts of the medial meniscus were removed, and the anatomical attachment site of the posterior horn was cleared. An aiming device was used for drilling a 2.4 mm tunnel from the anteromedial side of the tibia to the posterior horn location. After confirming the correct positioning, the 2.4 mm drill was overreamed with a 4.5 mm cannulated drill and threading was tapped in the first 2.5 cm of the tunnel to later receive the interference screws.

An initial estimation of the prosthesis size was made per‐operatively, and the corresponding size of a dedicated trial sizer instrument was inserted. After determination of the most suitable size, based on anatomical landmarks, two fixation tapes were looped through the horns of the meniscus prosthesis and a passing suture was used to pull the posterior horn of the prosthesis into place. Once more, correct sizing was confirmed, after which the optimal anterior horn location was determined in flexion and extension. Through the extended anteromedial portal, a 2.4 mm tunnel was drilled anterolaterally from the intended attachment site and over‐reamed to achieve a 4.5 mm tunnel. After it was prepared for the second interference screw, the anterior horn was also pulled into place with a passing suture.

The required amount of tension on the fixation tapes was determined in flexion and extension, to achieve adequate congruence with the knee joint anatomy and maintaining a full RoM. The interference screws were placed to lock the tensioned tapes in the bone tunnels. After a final arthroscopic check, the joint was rinsed, and the redundant parts of the tape outside the joint were cut.

### Rehabilitation

The rehabilitation programme allowed 50% weight bearing in the first 4 weeks after implantation and included RoM exercises with a physical therapist. After 4 weeks, knee joint swelling and the patient's walking pattern were assessed and, in consultation with the physical therapist, an increase of joint load towards 100% was allowed. RoM exercises were continued until full joint load and proper neuromuscular control in the index leg were achieved.

### Statistical analysis

For the change in PROMs between baseline and 12‐month follow‐up, mean and 95% confidence interval (CI) were determined with one‐sample *t*‐tests, and median and interquartile range were established using Tukey's hinges. A statistically significant change is indicated by the 95% CI not containing zero, assuming a normal distribution. All statistical analyses were performed using SPSS (version 29.0.1.0; IBM). Due to the small group size and the ordinal measurement level, only descriptive analyses were performed on the imaging outcomes.

## RESULTS

**Figure 3:**
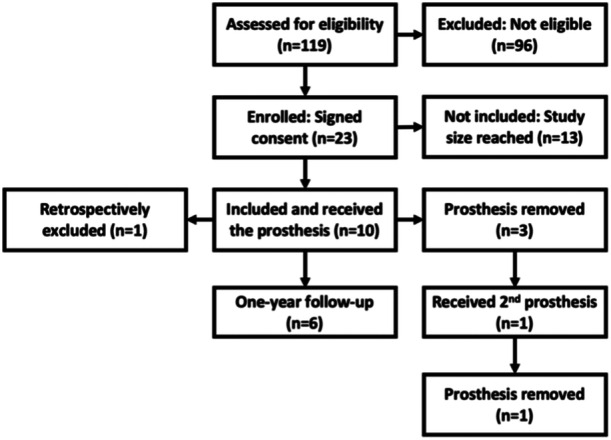
Study flowchart.

A total of 119 patients were screened, and out of the 23 eligible patients, 10 patients were included in the study (Figure 3). Baseline characteristics of all included patients are listed in Table 2. All patients had an unrestricted preoperative RoM, which was confirmed during surgery and not affected by implantation of the meniscus prosthesis. After prosthesis removal, patient 0301 received a second prosthesis within the study and was assigned a new case identifier (0308). Patient 0101 experienced several adverse events in the first months after implantation, including pain, swelling, wound leakage, and arthrofibrosis. Six months after implantation, it became clear that this patient had a low‐grade *Staphylococcus aureus* infection of the index knee. Retrospectively, this infection was found to be related to a previous shoulder surgery, which had commenced prior to the implantation of the meniscus prosthesis. As infection is one of the exclusion criteria (Table 1), patient 0101 has been retrospectively excluded from the analysis of study outcomes.

**Table 2:** Patient baseline characteristics.

Characteristic	Mean ± standard deviation (if applicable)		
	Overall	Survivors^a^	Failures
Number of patients^b^	9	6	3
Age (years)	42.0 ± 16.3	50.7 ± 12.0	24.7 ± 5.0
Male gender (*n*)	8	5	3
Weight (kg)	86.1 ± 16.1	83.0 ± 18.4	92.3 ± 10.2
Body mass index (kg/m^2^)	26.0 ± 2.4	25.7 ± 2.6	26.7 ± 2.2
Left index knee (*n*)	5	3	2
Time since last meniscectomy (months)	75.7 ± 71.5	92.2 ± 79.4	42.7 ± 47.3
Baseline KOOS Pain	49.7 ± 12.8	46.8 ± 13.8	55.6 ± 10.0
Cartilage status^c^ medial femoral condyle	1 (n = 4) 1/2 (n = 2) 2 (n = 3)	1 (n = 2) 1/2 (n = 1) 2 (n = 3)	1 (n = 2) 1/2 (n = 1)
Cartilage status^c^ medial tibial plateau	0 (n = 1) 1 (n = 6) 2 (n = 2)	1 (n = 4) 2 (n = 2)	0 (n = 1) 1 (n = 2)

Abbreviation: KOOS, knee injury and osteoarthritis outcome score.

^a^ Survivor: 1‐year follow‐up is reached.

^b^ Patient 0301/0308 is only considered once, patient 0101 is not included.

^c^ Modified Outerbridge classification, determined arthroscopically.

### Safety

The failure modes observed in the clinical study with the previous design of the meniscus prosthesis did not reoccur in this study: All of the implanted meniscus prostheses remained intact and the patients maintained their RoM. However, 4 out of 10 enroled patients did not reach 1‐year follow‐up due to the device‐related adverse events summarized in Table 3, which also includes the retrospectively excluded patient 0101.

**Table 3:** Summary of patients retrospectively excluded or not reaching 1‐year follow‐up.

Case ID	Last follow‐up	Δ KOOS Pain	Adverse events and remarks
0101	12 months	−16.7	Retrospectively excluded (infection); not considered in the analyses.
0102	3 months	−13.9	Explantation after 5 months, due to effusion, pain, suboptimal prosthesis position.
0208	6 months	30.6	Explantation after 8 months, due to dislocation after anterior and posterior tape failure.
0301^a^	3 months	0.0	Explantation after 4 months, due to posterior tape failure.
0308^a^	3 months	2.8	Reimplantation; explantation after 5 months due to posterior tape failure.

Abbreviation: KOOS, knee injury and osteoarthritis outcome score.

^a^ Cases 0301 and 0308 concern the same patient and the same knee joint.

Patient 0301 experienced a locking knee 3 months after implantation and MRI analysis revealed extrusion of the prosthesis. Arthroscopically, it was found that the meniscus prosthesis was intact, but the posterior tape had failed. The main reason for failure appeared to be a too‐anterior location of the posterior bone tunnel, likely caused by movement of the aiming device during drilling. Consequently, contact of the fixation tape with the medial femoral condyle resulted in wear and, ultimately, breakage of the tape. According to the protocol and after consultation with the patient, it was decided to replace the meniscus prosthesis and both fixation tapes, with an improved posterior horn location. The patient was followed up as if it was a new patient and received identifier 0308. Despite an improved fixation location, the posterior fixation tape failed again within the first 5 months, while the anterior tape and meniscus prosthesis were fully intact. The posterior tunnel location still turned out to be positioned too anteriorly and it was decided to remove the implants and fill the bone tunnels with donor bone. At the time of removal, pain scores and tibial cartilage were comparable to baseline, but the medial femoral cartilage had worsened from modified Outerbridge grade 1 to grade 2.

Two months after implantation, patient 0102 experienced a clicking sensation in the index knee, followed by pain and discomfort. Arthroscopy revealed that the meniscus prosthesis and fixation tapes were intact, but the posterior horn was positioned too far anteriorly and medially because of a malpositioned drill hole. This likely caused a subluxation of the posterior horn underneath the femoral condyle, explaining the clicking sensation and resulting in cartilage wear on the medial femoral condyle. Due to the extent of femoral cartilage damage, it was decided to remove the implants and that this patient was not eligible for reimplantation.

At the 6‐month follow‐up, patient 0208 reported strong clinical improvement, but shortly thereafter experienced locking, swelling, and pain in the knee joint shortly after. Eight months after initial surgery, both fixation tapes had failed, and the prosthesis was found in the suprapatellar pouch during arthroscopy. While the joint showed signs of light synovitis, the tibial and femoral cartilage status was comparable to baseline as evaluated by the modified Outerbridge score. The meniscus prosthesis and tapes were removed, but the interference screws were put back to maintain the bone tunnels for future options. No obvious causes for failure were identified, although in retrospect, implantation of a larger size might have been more suitable for this patient. It should be noted that patient 0208 was not fully compliant to the rehabilitation protocol, by not adhering to the restrictions regarding activity levels.

### PROMs

**Figure 4:**
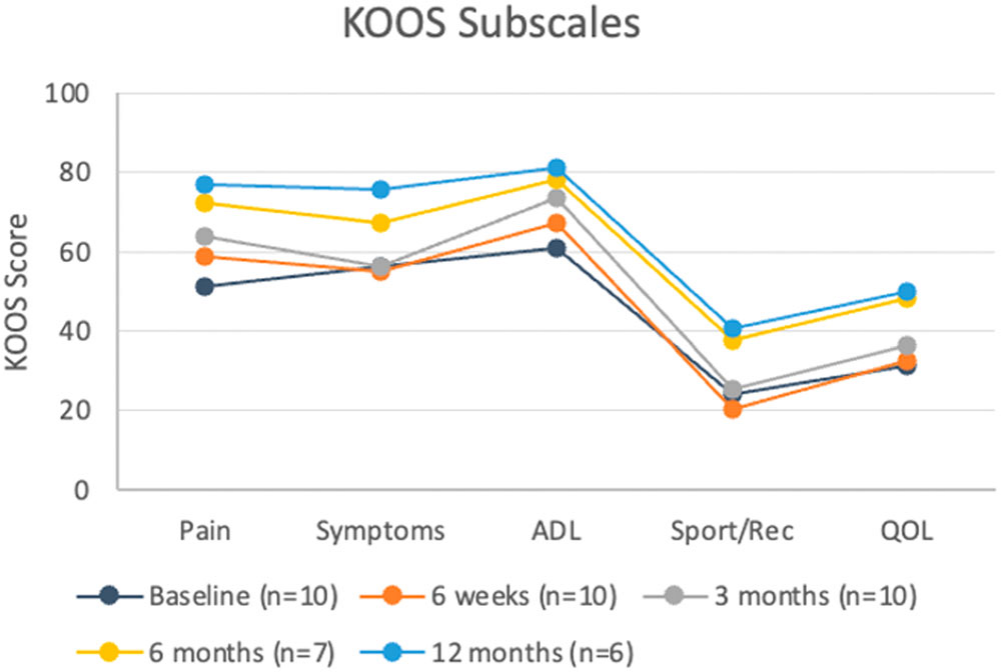
Mean KOOS scores after different follow‐up periods. Note that the number of patients is not the same for each timepoint. ADL, activities of daily living; KOOS, knee injury and osteoarthritis outcome score; QOL, quality of life.

**Figure 5:**
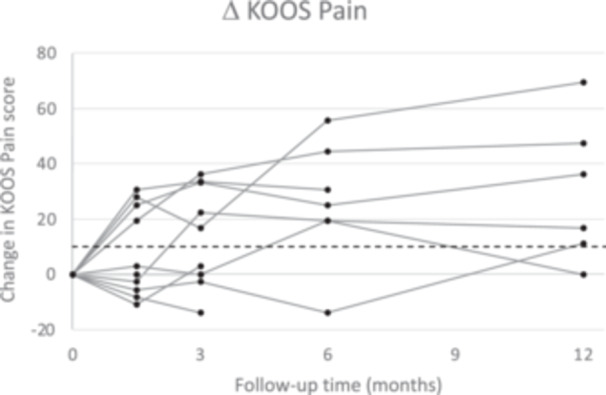
Difference in KOOS Pain score compared to baseline. The black dots and grey lines represent the individual cases, while the black dashed line indicates the minimal clinically important difference of 9.9 points. KOOS, knee injury and osteoarthritis outcome score.

All patients recovered well from surgery and PROMs already started to improve in the first 6 weeks (Figure 4). After 3 months, the mean improvement of the KOOS Pain subscale (12.8 points) surpassed the MCID of 9.9 points, although the standard deviation was large. At 6‐month follow‐up, three implants had been removed and the mean KOOS Pain improvement increased to 25.8 in the remaining patients. At 12‐month follow‐up, five out of six remaining patients reported an increase in KOOS Pain score exceeding the MCID (Figure 5 and Table 4). The mean improvement of 30.1 points was statistically significant, despite the large standard deviation of 25.8 points and a reduced group size (*p* < 0.05).

**Table 4:** Summary of change (Δ) in PROMs per patient after 12 months.

Case ID	KOOS Pain	KOOS Symp.	KOOS ADL	KOOS Sport	KOOS QOL	VAS Pain^a^	Lysholm	OKS	WORQ	EQ‐5D‐5L
0103	36.1	42.9	47.1	15.0	25.0	−56.0	12.0	3.0	25.0	3.0
0104	47.2	−3.6	51.5	35.0	31.3	−59.0	13.0	12.0	39.1	31.0
0204	69.4	39.3	55.9	85.0	37.5	−67.0	24.0	14.0	30.8	15.0
0205	16.7	17.9	14.7	5.0	−6.3	−15.0	0.0	13.0	13.5	0.0
0206	0.0	32.2	11.8	20.0	6.3	15.0	38.0	9.0	23.1	14.0
0207	11.1	3.6	−1.5	−25.0	−12.5	−9.0	−2.0	0.0	9.6	−5.0
**Mean**	**30.1**	**22.0**	**29.9**	**22.5**	**13.5**	**−31.8**	**14.2**	**8.5**	**23.5**	**9.7**
(95% CI)	(3.0, 57.1)	(1.9, 42.2)	(4.3, 55.5)	(−15.9, 60.9)	(−8.2, 35.3)	(−66.8, 3.2)	(−1.6, 30.0)	(2.5, 14.5)	(12.1, 34.9)	(−4.1, 23.4)
**Median**	**26.4**	**25.1**	**30.9**	**17.5**	**15.7**	**−35.5**	**12.5**	**10.5**	**24.1**	**8.5**
(IQR)	(11.1, 47.2)	(3.6, 39.3)	(11.8, 51.5)	(5.0, 35.0)	(−6.3, 31.3)	(−59.0, −9.0)	(0.0, 24.0)	(3.0, 13.0)	(13.5, 30.8)	(0.0, 15.0)
**MCID** b	**9.9**	**9.7**	**9.5**	**13.3**	**14.6**	**2.7**	**12.3**	‐	‐	‐

Abbreviations: ADL, activities of daily living; CI, confidence interval; EQ‐5D‐5L, 5‐level EQ‐5D health utility score; IQR, interquartile range; KOOS, knee injury and osteoarthritis outcome score; MCID, minimal clinically important difference; OKS, Oxford knee score; QOL, quality of life; Symp., symptoms; VAS, visual analogue scale; WORQ, work, osteoarthritis, and joint‐replacement questionnaire.

^a^ Higher VAS Pain scores indicate more pain, as opposed to the other outcome measures.

^b^ MCID [10, 19].

Table 4 further shows that all PROMs had improved at 12‐month follow‐up, both on average and for most of the patients. For KOOS Pain, KOOS Symptoms, KOOS ADL, OKS, and WORQ, the mean improvement compared to baseline was statistically significant (*p* < 0.05).

### Radiological outcomes

Table 5 shows the Kellgren–Lawrence OA classification scores, determined from radiographs, and modified Outerbridge cartilage grade, determined from MRI scans, at baseline and at 12‐month follow‐up. The MRI scans of three patients were performed at 6‐month follow‐up instead of 12‐month follow‐up, due to a protocol update after the first tape failures. Compared to the baseline, the modified Outerbridge grade improved for four out of six patients and remained stable for one patient, indicating that the affected articular surfaces did not deteriorate (Table 5).

**Table 5:** Imaging outcomes per patient.

Case ID	Osteoarthritis classification (Kellgren–Lawrence grade 0–4)		Medial cartilage status^a^ (modified Outerbridge grade 0–4)		Medial protrusion^b^ (mm)
	Baseline	12 months	Baseline	12 months	12 months
0103	1	1	2	3	7.5
0104	1	2	2	0^c^	1.8^c^
0204	2	2	2	2	8.1
0205	1	2	2	1^c^	7.9^c^
0206	1	1	1	0	6.7
0207	2	0	2	1^c^	3.7^c^

^a^ Only the highest grade out of the medial femoral condyle and medial tibial plateau is reported.

^b^ Defined as the distance between the medial edges of the prosthesis and the tibial plateau, measured in the coronal plane [4].

^c^ MRI at 6 months.

**Figure 6:**
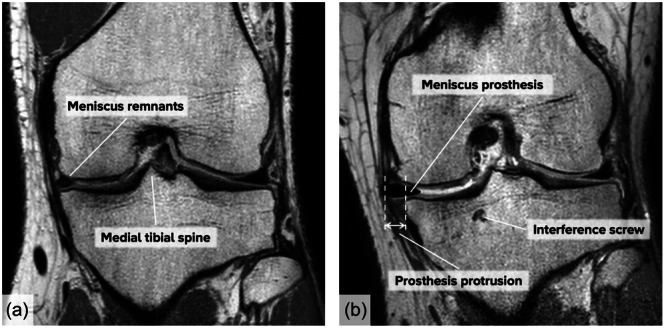
Typical example of (a) preoperative and (b) postoperative coronal MRI slices (patient 0103). The postoperative slice corresponding to the apex of the medial tibial spine was used to determine prosthesis protrusion [4]. MRI, magnetic resonance imaging.

Medial protrusion of the meniscus prosthesis (Figure 6) at follow‐up ranged from 1.8 to 8.1 mm, as also shown in Table 5. No baseline measurements could be obtained, as the baseline MRI scans were made preoperatively without prosthesis.

## DISCUSSION

The main finding of the present study was that none of the device‐related adverse events encountered during the first‐in‐human clinical study were reported for the updated design and fixation technique evaluated in the present study. Furthermore, the second generation of the medial meniscus prosthesis system may have the potential to treat postmeniscectomy pain, based on the promising clinical outcomes of the survivors after 12 months. However, the current configuration of the meniscus prosthesis system cannot yet be deemed safe for use in clinical practice, as new findings reveal the need to optimize the sizing and positioning of the prosthesis, reduce the wear rate of the tape, and subsequently increase the longevity of the fixation system.

In general, the orthopaedic surgeons expressed that the surgical procedure was feasible and went smoothly, but the survival of the posterior fixation tape appeared to be affected significantly by malpositioning of the posterior drill hole. Analyses of postoperative MRI scans and explanted meniscus prostheses and fixation tapes confirmed the likely association between the device‐related adverse events and the too‐anterior placement of the posterior drill holes.

Nevertheless, in patient 0208 both fixation tapes failed, and significant wear was observed, despite correct placement of the bone tunnels. The observed failures demonstrate that the amount of movement and friction, experienced by the tape when used for meniscus replacement fixation, may result in excessive wear and affect the longevity of the system.

In some patients, the prosthesis protruded considerably over the medial edge of the tibial plateau. A larger number of patients is needed to draw conclusions on any causality between prosthesis protrusion and clinical performance. Potential causes of this medial protrusion include oversizing, insufficient tensioning of the tapes during implantation, tape slippage, and tape breakage. Because no earlier postoperative MRI scans are available, it cannot be determined whether the prosthesis already protruded directly after implantation. Possible tape failure in these patients can, therefore, only be determined if additional surgery is required, as the tape is not clearly identifiable on MR images. Future studies should preferably include an MRI scan shortly after implantation surgery, to be able to evaluate prosthesis extrusion over time.

Improvement of positioning is expected to have a positive effect on the longevity of the meniscus prosthesis system, but the findings in patient 0208 demonstrate that this does not completely eliminate the risk of tape failure. Additional reduction of wear and failure risk may be achieved by preventing high tensile forces, excessive movement and friction, or by entirely changing the fixation design concept.

Preventing the overload of the tape may be achieved by restricting physical activity levels, which is not desirable, especially for younger patients. Implantation of a larger‐sized prosthesis is also likely to decrease the tensile forces on the tape, but care should be taken not to jeopardize clinical performance [7]. Preoperative prosthesis size selection, for example, based on MR imaging, may be of added value for future application.

Another method to improve the survival of the tape would be to limit its freedom of movement, as this would prevent wearing against the tibial surface. However, restriction of movement negates the advantages of tape fixation and reintroduces the chance of failure of the meniscus prosthesis itself [12, 21]. Although the meniscus prosthesis is not fixated peripherally, ‘bucket‐handle’‐like dislocation is not likely to occur due to its relative thickness and ability to move with the femoral condyle. The subluxation that occurred in patient 0102 does underline the importance of correct positioning especially the posterior fixation point.

Changing the direction of the drill tunnels may decrease the angle of the tunnel exit and avoid the so‐called ‘killer angle’, potentially reducing the cutting and wearing effect. By choosing a different material or manufacturing technique, the friction coefficient, wear characteristics and tensile strength of the tapes could potentially be improved. Unfortunately, no performance data of different sutures and tapes is available for this application [23]. With any changes to the devices and surgical technique, new risks might be introduced, and potential adverse effects should be investigated. Therefore, further preclinical and clinical research should determine the optimal combination of construct, material, manufacturing technique and surgical procedure.

The primary clinical performance endpoint was the change in patient‐reported KOOS Pain score at the 1‐year follow‐up. KOOS Pain scores significantly improved for the patients reaching 1‐year follow‐up (mean improvement of 30.1 points) and resulted in five out of six patients achieving a positive MCID. The mean change in KOOS Pain after 1 year found in this study is comparable to the value reported for the NUsurface® implant (30.4 points) and MAT (22–25 points) [1, 11, 15]. Another study found that 64% of patients achieved the MCID in KOOS Pain 1 year after MAT, a percentage that remained constant the year after [3].

The clinical performance is evaluated in combination with safety, i.e., potential disadvantages and adverse events, to enable a risk‐benefit analysis. Implantation of a NUsurface® meniscus replacement results in an 18% reoperation after 1 year [26]. For MAT as a surgical treatment option for postmeniscectomy pain syndrome, reoperation rates of 20% after 24, and 59% after 43 months were reported [1, 16]. Unfortunately, no reoperation rate within the first year could be deduced from these studies. Nevertheless, it does reveal that the risk‐benefit ratio of this treatment is often described as favourable even though more than half of the patients are subjected to a revision transplantation or meniscectomy within 4 years [16, 17, 25]. The fixation method of the meniscus prosthesis requires a redesign to significantly reduce the current reoperation rate of 40% in the first year after implantation. The lowest possible failure rate should be pursued, although the use of implants always comes with the risk of infection and other complications [11, 16, 18].

On a group level, the results of this study do not demonstrate a worsening of cartilage condition after implantation of the meniscus prosthesis. Although the follow‐up time is relatively short and the group size is too small to draw any hard conclusions, no adverse effects on the articular cartilage status or KOOS Pain score were found, even in most cases of tape failure. This finding means that a device failure should not necessarily be considered a treatment failure, as the failed implants can be replaced relatively easily during a second arthroscopic procedure. Furthermore, other surgical interventions such as osteotomy, total knee arthroplasty, or even MAT remain possible after treatment with the meniscus prosthesis.

Due to the small group size, no conclusions can be drawn from the comparison between survivors and failures in Table 2, with regard to predictive factors for clinical and mechanical failure. However, the failures seem to be somewhat younger and heavier than the survivors. One might imagine that younger patients with less pain are more active and expose the joint to higher forces. Higher body weights might further increase the chance of knee overloading and implant failure. After extensive preclinical evaluation, larger clinical studies should provide more insight into the optimal inclusion criteria for treatment with the meniscus prosthesis, including boundary conditions for joint loading and anatomy.

These future studies will include a control group and aim to evaluate the safety and clinical outcomes of the meniscus prosthesis system with a new fixation method. When the device failure rate is reduced and the possibility for alternative treatment options is maintained, the next generation of the meniscus prosthesis system is expected to be a beneficial solution for patients with postmeniscectomy pain syndrome.

## CONCLUSION

The failure mechanisms that occurred with the previous design have been resolved, but the new fixation technique introduced new safety issues. Improvement of positioning and fixation techniques are considered essential future adaptations to reduce the risk of failure. The good clinical outcomes reported by the patients reaching 1‐year follow‐up indicate that the medial meniscus prosthesis is a potential solution for patients suffering from postmeniscectomy pain syndrome.

## AUTHOR CONTRIBUTIONS

Branco S. van Minnen, Thijmen Struik, Sebastiaan A. W. van de Groes, Nico Verdonschot and Tony G. van Tienen designed the meniscus prosthesis system. Tony G. van Tienen was involved in designing the clinical study. Petra J. C. Heesterbeek, Koen C. Defoort, Pieter J. Emans, Ewoud R. A. van Arkel, Liesbeth M. Jutten and Saskia Susan included all patients in the study. Koen C. Defoort, Pieter J. Emans, Ewoud R. A. van Arkel and Tony G. van Tienen performed the surgical procedures. All authors were involved in interpreting the study outcomes. Branco S. van Minnen drafted the manuscript. All authors commented on previous versions of the manuscript and approved the final manuscript.

## CONFLICTS OF INTEREST STATEMENT

B. S. van Minnen and T. Struik are employed at ATRO Medical BV. B. S. van Minnen, T. G. van Tienen and T. Struik own stocks in ATRO Medical BV. B. S. van Minnen, T. G. van Tienen and T. Struik are inventors of applicable patents but have no ownership. T. G. van Tienen receives a management fee from ATRO Medical BV. The Institute of N. Verdonschot receives a consultation fee from ATRO Medical BV. The remaining authors declare no conflict of interest.

## ETHICS STATEMENT

The medical ethical committee METC Oost‐Nederland and competent authorities approved this study, which is registered in the Dutch Trial Register under NTR ID NL9805 and CCMO ID NL75393.000.21. Written informed consent was obtained from all individuals participating in the study.

## Data Availability

Research data will not be shared until after the study's completion.
